# Identifying future geriatric nurses: psychometric evaluation of the German Willingness to Work with Elderly People Scale among nursing students

**DOI:** 10.3389/fpsyg.2026.1933160

**Published:** 2026-10-15

**Authors:** Shengze Zhi, Burcu Akpınar Söylemez, Jiao Sun, Birgit Teichmann

**Affiliations:** 1 School of Nursing, Jilin University, Changchun, Jilin, China; 2 Network Aging Research, Heidelberg University, Heidelberg, Germany; 3 Department of Internal Medicine Nursing, Faculty of Nursing, Dokuz Eylul University, Izmir, Türkiye

**Keywords:** aged, attitude, geriatric nursing, nursing students, psychometrics, validation study, workforce

## Abstract

**Introduction:**

In German-speaking countries, where geriatric care faces a persistent nursing shortage, nursing students are expected to play a key role in addressing future workforce needs. Currently, there is a lack of valid and reliable instruments to assess nursing students’ willingness to work with older adults. This cross-sectional study aimed to validate the German version of the Willingness to Work with Elderly People (WEPS-D) Scale and examine factors associated with the willingness to work with older adults to support geriatric workforce development in nursing education.

**Methods:**

A convenience sample of 207 German-speaking nursing students completed the survey, including 52 students who participated in a 4-week retest. Structural validity was evaluated using exploratory factor analysis (EFA), while convergent validity was assessed through correlations with the Kogan Attitudes toward Older People Scale (KAOP), Age Stereotypes (AS), and Future Self-views (FS). Internal consistency and test–retest reliability were examined. In addition, multiple linear regression was used to identify factors independently associated with willingness to work with older adults.

**Results:**

EFA identified a four-factor structure (intention, perceived behavioral control, attitude, and subjective norm) with 20 items, explaining 49.01% of the total variance. Convergent validity was supported at the factor level, with factor 3 (Attitude) showing a moderate correlation with the KAOP (*r* = 0.515, *p* < 0.01), while the total WEPS-D score showed a lower correlation (*r* = 0.385, *p* < 0.01) that did not meet the pre-specified criterion. At the scale level, internal consistency (Cronbach’s α = 0.894, McDonald’s ω = 0.902) and test–retest reliability (ICC = 0.882) were good, although factors 3 (Attitude) and 4 (Subjective norm) showed relatively low internal consistency. Multiple linear regression showed that higher education and negative or neutral attitudes toward aging were significantly associated with lower WEPS-D scores compared with vocational training and positive attitudes, respectively.

**Conclusion:**

The WEPS-D is a valid and reliable tool for assessing nursing students’ willingness to work with older adults. It can be used in schools to identify the potential workforce in geriatric nursing and guide early educational interventions.

## 1 Introduction

Population aging is one of the most significant demographic transformations worldwide. With decreasing birth rates and longer life expectancies, populations across the globe are aging rapidly (Khan et al., 2024). According to the German Federal Statistical Office, people aged 65 years and older accounted for 23.2% of the total population in 2024 (Statistisches Bundesamt (Destatis), 2025), indicating that Germany has already surpassed the threshold of an aged society, defined as having more than 20% of its population aged 65 or above (Economic and Social Commission for Asia and the Pacific, 2017). As populations age, the prevalence of chronic diseases, multimorbidity, functional decline, and long-term care needs increases substantially, placing considerable pressure on healthcare and long-term care systems (Khan et al., 2024; Statistisches Bundesamt (Destatis), 2026).

To address these challenges, a sufficiently prepared nursing workforce is essential. Nurses constitute the largest occupational group within the healthcare workforce and represent an indispensable force in playing a central role in promoting healthy aging, supporting functional independence, and providing person-centered care for older adults (World Health Organization, 2025). However, Germany continues to face substantial shortages in the nursing workforce, particularly within geriatric and long-term care settings. In 2025, approximately 31,000 nursing vacancies were registered with the Federal Employment Agency in Germany (Federal Employment Agency Statistics, 2026). Given the increasing demand for elderly care services, attracting future nurses to work with older adults has become an important educational and workforce priority (Momtaz et al., 2018).

Nursing students represent the future geriatric workforce. Their willingness to work with older adults may influence future career choices and, consequently, affect the availability of qualified professionals in geriatric care. Previous research has demonstrated that many nursing students prefer acute care or highly specialized clinical fields, while geriatric nursing is often perceived as less attractive (Dai et al., 2021). Understanding students’ willingness to work with older adults and the factors influencing this willingness is therefore important for nursing educators seeking to strengthen recruitment into geriatric care.

Educational institutions require reliable instruments to identify students’ career preferences and evaluate interventions designed to improve interest in geriatric nursing. In German-speaking countries, several instruments have been utilized to assess attitudes and perceptions toward aging and older adults. The Aging Semantic Differential (ASD), developed by Rosencrantz and McNevin in 1969 (Rosencranz and McNevin, 1969), is a 32-item self-report instrument using pairs of bipolar descriptors, such as “Progressive—Old-fashioned”. However, the ASD can be applied to assess attitudes across different age groups rather than specifically focusing on older adults. In adition, the Kogan Attitude toward Older People (KAOP) (Kogan, 1961), as well as Age Stereotypes (AS) and Future Selves (FS) (Kornadt et al., 2020) were originally developed to assess attitudes, perceptions, and expectations regarding older adults and aging. Nevertheless, these instruments are primarily designed for use in the general population and do not specifically focus on nursing students or incorporate profession-related perspectives and knowledge relevant to geriatric nursing. Moreover, these instruments pay limited attention to behavioral intentions regarding working with older adults.

The Willingness to Work with Elderly People (WEPS) Scale, developed by Momtaz et al. (2018), was specifically designed to assess medical and nursing students’ willingness to work with older adults (Momtaz et al., 2018). Based on the theory of planned behavior, the scale proposed four subdimensions: attitude, subjective norm, and perceived behavioral control. The WEPS has subsequently undergone cultural adaptation and psychometric validation in Türkiye (Akpınar Söylemez et al., 2022) and China (Unpublished manuscript, 2026). To date, no validated German version of the WEPS (WEPS-D) is available, representing a significant research gap in the German context.

In addition to measurement issues, relatively little is known about which characteristics of German-speaking nursing students are associated with willingness to work with older adults. Clement and Krueger (2002) described the process of social projection, whereby individuals project their own experiences onto generalized perceptions of others, influencing individuals’ perceptions and responses toward specific social groups, including older adults (Clement and Krueger, 2002). Previous studies conducted in Finland and Austria have shown that the demographic characteristics and personal experience of nursing students were associated with the perceptions of aging and older people (Salin et al., 2020; Schüttengruber et al., 2022). For example, nursing professionals with more than 5 years of work experience, as well as those having close personal contact with older adults, demonstrated a more positive attitude and perceptions toward older adults compared with those with limited or no experience. Given that personal experiences may shape individuals’ views of older adults, it is possible that such experiences may also influence nursing students’ willingness to work with older adults. However, this relationship remains underexplored, particularly in Germany. Identifying such factors may help nursing educators develop targeted educational strategies to strengthen students’ interest in working with older adults.

Therefore, the objectives of this study were (1) to culturally adapt and psychometrically evaluate the WEPS-D among nursing students and (2) to explore the demographic and experiential factors of nursing students associated with willingness to work with older adults. This study seeks to provide nursing educators with a valid tool to support geriatric workforce development and educational planning.

## 2 Materials and methods

### 2.1 Study design

This cross-sectional study followed the STrengthening the Reporting of OBservational studies in Epidemiology (STROBE) guidelines (von Elm et al., 2007) (Supplementary Table S1) and employed a two-phase design consisting of cross-cultural adaptation and psychometric validation. A second round of data collection was conducted 4 weeks later to assess test–retest reliability.

### 2.2 Cross-cultural adaptation phase

The translation and adaptation were conducted in accordance with Guidelines for the Process of Cross-Cultural Adaptation of Self-Report Measures (Beaton et al., 2000). First, two independent native German speakers translated the original English version into German. Any discrepancies in the translation process were discussed by the research team, and then, Synthesis I was produced. Second, Synthesis I was back-translated by an English translator. The back-translation was then compared with the original version and discussed to generate Synthesis II. Third, an expert panel of five members (two gerontologists, two psychologists, and one nurse) evaluated the response methods, sentence formats, wordings, grammatical structures, and content of each item in Synthesis I and Synthesis II and gave some feedback. Each item was rated on a four-point scale ranging from 1 (not appropriate at all) to 4 (completely appropriate). Content validity was assessed using the content validity index (CVI) (Polit et al., 2007). Any necessary modifications were made in accordance with the experts’ suggestions, leading to Synthesis III. Fourth, a pilot test was performed with five nursing students to ensure the comprehensibility and feasibility of the scale. The final German version of the WEPS (WEPS-D) is publicly available on the Open Science Framework^1^.

### 2.3 Validation phase

#### 2.3.1 Participants

The study focused on nursing students because they represent the future workforce in geriatric care. Understanding their willingness to work with older adults is particularly relevant for nursing education and workforce planning in aging societies. According to the principle of factor analysis procedure, a sample size of at least 5–10 participants per item is recommended (Shultz et al., 2020). Inclusion criteria were (a) being a nursing student at a German-speaking nursing school and (b) being aged 16 years or older, consistent with the minimum age in German-speaking countries after completion of compulsory schooling (European Centre for the Development of Vocational Training (Cedefop), 2022), with sufficient cognitive and judgmental capacity to understand the study information. The exclusion criterion was prior participation in the pilot or translation process of this scale. Finally, a total of 228 questionnaires were collected. After excluding students from other majors and questionnaires with incomplete or invalid data, 207 nursing students were included in the final analysis.

#### 2.3.2 Data collection

Data collection took place from January 2026 to April 2026. An online survey was conducted using a convenience sampling method. The questionnaire was hosted on the widely used German online survey platform, SoSci Survey^2^. Participants were recruited with the assistance of educational institutions. The researchers contacted faculty members at German-speaking nursing schools and universities via email and invited them to support participant recruitment. Faculty members then distributed the electronic survey link to nursing students through internal communication channels. Students could complete the questionnaire on mobile devices or computers in approximately 15 min. Approval to conduct the study was obtained from the Ethics Committee of the Faculty of Behavioral and Empirical Cultural Sciences, Heidelberg University (AZ Tei2025 2/1). All students who agreed to participate in the study provided electronic consent.

#### 2.3.3 Demographic characteristics

Based on the existing literature, demographic characteristics were assessed: gender, age, country, education level, and marital status. In addition, experiences and perceptions related to older adults were evaluated, including the presence of older adults in the neighborhood, whether participants still had grandparents, the living arrangements of their closest grandparents, the frequency of contact with grandparents, previous experience working with older adults, prior visits to nursing homes or geriatric clinics, perceptions regarding the age at which a person is considered old, willingness to work in nursing homes, geriatric care, or geriatric medicine in the future, preferred age groups for future work, major concerns regarding aging, the individuals who most strongly influenced participants’ views of old age, attitudes toward aging, and subjective age perception compared with their actual chronological age.

#### 2.3.4 Instruments

##### 2.3.4.1 WEPS

The WEPS consists of 20 items, comprising the following four dimensions (Momtaz et al., 2018): attitude, subjective norm, perceived behavioral control, and intention. Responses are rated on a six-point Likert scale, ranging from 1 (strongly disagree) to 6 (strongly agree). Items 2, 3, 4, and 17 are reverse-coded. The total score is obtained by summing all item scores, ranging from 20 to 120, with higher scores indicating a stronger willingness to work with older people. The original language of the scale is English. The Cronbach’s α coefficient of the original scale was 0.899 (Momtaz et al., 2018).

##### 2.3.4.2 KAOP

The KAOP scale was developed by Nathan Kogan in 1961 (Kogan, 1961) to assess the attitudes of nursing staff or students toward older adults and was translated and validated in German by Teichmann et al. (2026). It originally consists of 16 negative items and 16 positive items. Responses are rated on a six-point Likert scale, ranging from 1 (strongly agree) to 6 (strongly disagree). After reverse-scoring the negative items, the total scale score ranges from 32 to 192, with higher scores indicating a more positive attitude toward older adults. A Cronbach’s α of 0.87 is shown for the German version (Teichmann et al., 2026).

##### 2.3.4.3 AS and FS

The brief version of AS and FS, which assess individuals’ views of older adults and their expectations regarding their own future life in old age, was developed by Anna E. Kornadt, Thomas M. Hess, and Klaus Rothermund (Kornadt et al., 2020). It contains nine domains (“Family”, “Friendships”, “Leisure”, “Personality”, “Finances”, “Work”, “Appearance”, “Fitness”, and “Health”). Each domain uses a bipolar eight-point Likert format, ranging from 1 (less positive evaluation) to 8 (more positive evaluation), with higher scores reflecting more positive views on aging. For each domain, AS were assessed before current FS.

#### 2.3.5 Statistical analysis

The statistical analyses were conducted via IBM SPSS 31.0 and R version 4.6.1.

##### 2.3.5.1 Preliminary item statistics

The Kolmogorov–Smirnov test was utilized to assess the normality of each item and the total score. Depending on the data distribution, Spearman’s or Pearson’s correlation coefficients were calculated to examine the correlations between individual item scores and the total score. Correlation coefficients > 0.40 indicated good inter-total correlations within the scale. Items with correlation coefficients < 0.20 or correlations that did not reach statistical significance were considered for deletion (Wu, 2010).

##### 2.3.5.2 Structure validity

To examine the underlying factor structure of the WEPS-D, an EFA using principal axis factoring (PAF) was performed. Given the theoretical correlations among the dimensions of the WEPS, an oblique Promax rotation was applied (Watkins, 2018). The suitability of the data for factor analysis was assessed using the Kaiser–Meyer–Olkin (KMO) measure and Bartlett’s test of sphericity (Wu, 2010). The KMO values between 0.8 and 1.0 were considered superb, and Bartlett’s test of sphericity should be significant (*p* < 0.05). Initial factor extraction was guided by Kaiser’s criterion (eigenvalues > 1) and inspection of the conventional scree plot (Feng, 2015). Parallel analysis was also conducted by comparing observed eigenvalues with those obtained from randomly generated data under the same sample size and number of variables (Horn, 1965). Compared with the conventional Kaiser criterion of retaining factors with eigenvalues greater than 1, parallel analysis takes sampling error into account and has been recommended as a more robust approach for determining the number of factors (Mills et al., 2014). The factor correlation matrix was also examined to evaluate the adequacy and interpretability of the factor solution. Inter-factor correlations above 0.80–0.85 may indicate poor discriminant validity and warrant further examination of factor distinctiveness (Brown, 2015). The final number of factors was determined by considering the eigenvalue criterion, scree plot, parallel analysis, factor correlation matrix, and theoretical interpretability of the factor solution. For the oblique Promax rotation, factor loadings were interpreted using the pattern matrix. The coefficients in the pattern matrix, referred to as pattern coefficients, represent standardized partial regression coefficients rather than simple factor-item correlations and may theoretically exceed |1.00| (Watkins, 2018). Pattern coefficients of ≥ 0.30 were considered minimally acceptable (Hair et al., 2019).

##### 2.3.5.3 Convergent validity

Convergent validity of the WEPS-D was assessed by examining correlations with three established self-report scales (Zhang and Zhou, 2020): the KAOP, AS, and FS. These instruments were selected because they assess theoretically related constructs such as attitudes toward older adults, age stereotypes, and perceptions of aging. Because AS and FS assessed perceptions across nine distinct life domains, respectively, correlations were calculated between the WEPS-D score and each domain score separately rather than using an overall total score. Correlation coefficients between 0.4 and 1.0 were considered indicative of moderate to high convergent validity, supporting the scale’s ability to measure the intended constructs (Zhang and Zhou, 2020).

##### 2.3.5.4 Test–retest reliability

For test–retest reliability, the evaluation followed the intraclass correlation coefficient (ICC) standards, where values < 0.40 were considered poor, 0.40–0.60 as fair, 0.60–0.75 as good, and 0.75–1.0 as excellent (Cicchetti, 1994). Test–retest reliability was assessed using a two-way mixed-effects model with an absolute-agreement definition for single measurements, and ICC estimates were reported with 95% confidence intervals (CI) (Koo and Li, 2016). Assuming ICC = 0.6, *β* = 0.20, α = 0.05, and two observations per participant, the required sample size was 15 participants (Bujang and Baharum, 2017). Considering a potential attrition rate of 25%, at least 20 participants were needed for the test–retest reliability analysis. A 4-week interval was used between the two assessments (Teichmann et al., 2025) to ensure stability of responses over time. Participants were asked to create a personal code so that data from the two surveys could be matched. In addition, participants were asked to provide their email to receive the link for the second assessment.

##### 2.3.5.5 Internal consistency

Cronbach’s α was used to evaluate the internal consistency of the WEPS-D, with values of 0.70–0.95 indicating good internal consistency (Shultz et al., 2020; Terwee et al., 2007). McDonald’s ω was also calculated because it accounts for varying unstandardized pattern coefficients and error variances (McDonald, 2013). If the factor had two items, the Spearman-Brown coefficient was reported because it is considered an appropriate reliability estimate for two-item scale (Eisinga et al., 2013).

##### 2.3.5.6 Floor and ceiling effects

Floor and ceiling effects were additionally examined and considered when more than 10% of participants achieved the lowest or highest possible score on the scale (Terwee et al., 2007).

##### 2.3.5.7 Correlation analyses

Univariate analyses were conducted to examine associations between demographic variables and willingness to work with older adults. Independent *t*-tests or Mann–Whitney U tests were used for dichotomous variables, while one-way analysis of variance (ANOVA) or Kruskal–Wallis tests were used for variables with more than two categories (Feng, 2015). For statistically significant one-way ANOVA results, Bonferroni-adjusted *post-hoc* pairwise comparisons were performed to account for multiple comparisons (Kim, 2015). Notably, multiple-choice items were summarized descriptively only and were not included in inferential analyses because the response categories were not mutually exclusive. Variables with *p* < 0.05 in univariate analyses were entered into a multiple linear regression model to identify factors independently associated with willingness to work with older adults (Feng, 2015).

## 3 Results

### 3.1 Participant characteristics

Of the 207 German-speaking nursing students, 170 were female students (82.1%), and the mean age was 26.81 ± 9.94 years (Table 1). Thirteen students (6.3%) were aged 16–17 years. Most students were German (85.5%), completed vocational training (61.8%), and were single (80.7%). There were 196 students (94.7%) who reported having older adults living in their neighborhood, while 121 students (58.5%) had grandparents. Many students lived far away from their grandparents (*n* = 65) and met them several times a year (*n* = 41). Almost all students worked with older adults in their profession or job (98.6%) or visited a nursing home or a geriatric clinic (88.4%).

**Table 1:** Characteristics of nursing students (*N* = 207).

Characteristics	Nursing students		WEPS-D	*r/t/F*	*P*
	*n* (m)	% (SD)	m (SD)		
Participant characteristics					
Gender				*t* = −0.783^a^	0.435^a^
Male	35	16.9	85.51 (10.79)		
Female	170	82.1	88.03 (15.84)		
Diverse	2	1.0	92.50 (0.71)		
Age^e^	26.81	9.94	87.65 (15.04)	*r* = 0.175	**0.012**
Country				*t* = 1.307^b^	0.193^b^
Germany	177	85.5	88.21 (15.09)		
Austria	27	13.0	82.89 (14.19)		
Others	3	1.4	97.33 (12.85)		
Education				*t* = 2.675^c^	**0.008** ^c^
Vocational training	128	61.8	89.81 (14.94)		
Undergraduate studies	55	26.6	84.67 (13.41)		
Graduate studies	7	3.4	78.28 (13.12)		
Dual studies	14	6.8	85.00 (17.53)		
Others	3	1.4	84.00 (28.83)		
Marital status				*t* = −1.754^d^	0.081^d^
Single	167	80.7	86.75 (14.17)		
Married	37	17.9	91.54 (18.28)		
Divorced	2	1.0	81.00 (11.31)		
Widowed or deceased partner	1	0.5	106		
Experience with older adults					
Do older adults live in the neighborhood?				*F* = 3.890	**0.022**
No	11	5.3	80.00 (9.81)		Several vs. No: *p* = 0.094;
Yes, several	140	67.6	89.52 (14.58)		Several vs. Only a few: *p* = 0.125;
Yes, only a few	56	27.1	84.45 (16.18)		No vs. Only a few: *p* = 1.000
Do you still have grandparents?				*t* = 1.394	0.165
No	86	41.5	89.37 (16.19)		
Yes	121	58.5	86.42 (14.11)		
Where do your closest grandparents live?^#^				——	——
In the same house	19	9.2	95.16 (13.10)		
In the same neighborhood	13	6.3	88.23 (11.35)		
In the same town/city	31	15.0	84.26 (13.86)		
In another nearby place	42	20.3	89.26 (13.82)		
In a nearby nursing home	7	3.4	87.86 (14.50)		
In a distant nursing home	10	4.8	80.90 (13.88)		
Far away	65	31.4	83.14 (15.39)		
How often do you see the grandparents you see most frequently?^#^				——	——
Daily	14	6.8	98.14 (12.88)		
Several times a week	16	7.7	92.06 (10.09)		
Once a week	13	6.3	84.84 (16.32)		
Several times a month	30	14.5	88.33 (15.61)		
Once a month	22	10.6	84.27 (12.78)		
Several times a year	41	19.8	85.31 (16.68)		
Once a year	16	7.7	82.68 (17.13)		
Less than once a year	9	4.3	80.44 (12.84)		
I have no contact with my grandparents	13	6.3	82.53 (15.75)		
Have you already worked with older adults in your profession or job?				*t* = −0.388	0.698
No	3	1.4	91.00 (17.52)		
Yes	204	98.6	87.60 (15.05)		
Have you ever visited a nursing home or a geriatric clinic?				*t* = −0.179	0.861
No	22	10.6	88.18 (17.76)		
Yes	185	88.4	87.58 (14.74)		
Perceptions of older adults					
At what age do you think a person is considered old? ^#^				——	——
Beginning of retirement	51	24.6	88.12 (15.89)		
When a person has gray hair	2	1.9	84.75 (16.74)		
When a person no longer understands humor	9	4.3	95.55 (10.35)		
When a person has wrinkles	1	0.5	61.00		
From 40 years of age	1	0.5	109.00		
From 50 years of age	4	1.9	97.00 (13.29)		
From 60 years of age	37	17.9	88.73 (14.93)		
From 65 years of age	48	23.2	88.75 (15.44)		
From 70 years of age	69	33.3	86.43 (14.08)		
From 75 years of age	39	18.8	88.15 (14.34)		
From 80 years of age	34	16.4	89.71 (15.38)		
From 85 years of age	13	6.3	95.38 (12.97)		
From 90 years of age	11	5.3	97.18 (13.38)		
Can you imagine working in a nursing home, geriatric care, or geriatric medicine in the future? ^#^				——	——
No	70	33.8	75.00 (12.62)		
Yes	84	40.6	98.82 (9.71)		
Maybe	57	27.5	87.33 (11.17)		
With which age group would you most like to work? ^#^				——	——
Newborns/infants (birth to 1 year)	52	25.1	80.42 (15.31)		
Children (2–12 years)	47	22.7	78.91 (15.81)		
Adolescents (13–18 years)	42	20.3	80.26 (15.37)		
Young adults (19–39 years)	70	33.8	81.80 (14.26)		
Middle-aged adults (40–59 years)	73	35.3	85.46 (14.11)		
Older adults (60 years and older)	69	33.3	97.41 (11.91)		
Mixed-age groups	81	39.1	88.90 (12.77)		
It does not matter to me	24	11.6	96.58 (9.55)		
Which topics concern you most regarding aging? ^#^				——	——
Health	172	83.1	88.04 (15.07)		
Loneliness	114	55.1	86.02 (14.95)		
Financial insecurity	132	63.8	85.30 (14.62)		
Dependence on care	115	55.6	85.64 (15.97)		
Abuse/discrimination	28	13.5	92.29 (14.09)		
Others	5	2.4	84.00 (12.19)		
Who has influenced your view of old age the most? ^#^				——	——
Grandmother	101	48.8	89.34 (15.10)		
Grandfather	58	28.0	85.91 (14.47)		
Other person	47	22.7	86.70 (15.07)		
No one	22	10.6	85.00 (18.08)		
I do not know	53	25.6	84.41 (12.83)		
What is your attitudetoward aging?				*F* = 16.298	**< 0.001**
Positive	78	37.7	94.56 (12.69)		Positive vs. Negative: *p* < 0.001;
Negative	19	9.2	79.05 (16.23)		Positive vs. Neutral: *p* < 0.001;
Neutral	110	53.1	84.22 (14.53)		Negative vs. Neutral: *p* < 0.418
Compared with your current chronological age, how old do you feel?				*F* = 1.044	0.386
Much younger	33	15.9	88.06 (15.94)		
Somewhat younger	48	23.2	87.48 (14.64)		
About my actual age	71	34.3	89.53 (14.31)		
Somewhat older	45	21.7	83.96 (15.62)		
Much older	10	4.8	90.30 (16.26)		

#: Multiple Choice; a: Gender was analyzed as non-female versus female. Participants identifying as “Diverse” were combined with “Male” due to the small sample size. b: Country was analyzed as Germany versus Austria/Others. Participants identifying as “Others” were combined with Austria due to the small sample size. c: Education was analyzed as vocational training versus higher education. Participants in “Graduate studies”, “Dual studies”, and “Others” were combined with undergraduate studies due to the small sample sizes. d: Marital status was analyzed as single versus married. Divorced and widowed participants were combined with married participants due to the small sample sizes. e: Age values were presented as mean (SD); *r* = Pearson’s correlation coefficient; *t* = independent-samples *t*-test statistic; F = one-way analysis of variance (ANOVA) statistic. Bold values indicate statistically significant differences or associations (*p* < 0.05).

### 3.2 Content validity

Five experts assessed the content validity. The item-level CVI (I-CVI) range of this scale was 0.8 to 1.0. The scale-level CVI (S-CVI), calculated using the averaging method, was 0.97, indicating excellent overall content validity. Interrater agreement was assessed using Kendall’s coefficient of concordance, yielding a statistically significant level of agreement among experts (Kendall’s W = 0.62, *p* < 0.001).

### 3.3 Preliminary item statistics

The correlation coefficient between the score of each item and the total score of the scale ranged from 0.223 to 0.875 (*p* < 0.001) (Table 2). Items 2, 4, 6, and 13 showed correlations between 0.20 and 0.40, which were considered acceptable.

**Table 2:** Item-level item-total correlations and pattern coefficients of the four-factor solution (*N* = 207).

Items		Factor 1	Factor 2	Factor 3	Factor 4	Item-total correlation
1	Working with the elderly is gratifying	0.501				0.730*
2	Care of elderly patients is the waste financial resources^#^			0.555		0.223*
3	Working with aged patients is disappointing^#^			0.490		0.622*
4	Working with older adults is a dead-end job^#^			0.613		0.281*
5	Working with the elderly is a highly rewarding experience			0.314		0.487*
6	Caring for the elderly is a human duty				0.618	0.371*
7	My culture encourages me to work with the elderly				0.692	0.466*
8	There is enough encouragement for working with the elderly	0.568				0.568*
9	My professors advise me to consider aged care career		0.409			0.573*
10	Working with elderly people is socially valuable			0.311		0.493*
11	I achieve competencies for the care of older patients		0.367			0.541*
12	I have professional competencies in aged care		0.843			0.423*
13	In the curriculum there is enough aged care training		0.345			0.354*
14	I have the skills to work with the elderly		0.751			0.578*
15	I have the necessary capabilities to provide end-of-life care		0.686			0.494*
16	One of my career priorities upon graduation is aged care	0.757				0.875*
17	I will never consider an aged care job^#^	0.562				0.673*
18	After graduation, I would like to work in community aged care	0.967				0.806*
19	Upon graduation, I would prefer to work with the elderly	1.032				0.833*
20	After graduation, I definitely choose working with older people	0.983				0.833*

*p < 0.01. Blank cells indicate pattern coefficients < 0.30 in the pattern matrix. ^#^Items 2, 3, 4, and 17 were reverse-scored before calculating item-total correlations and pattern coefficients.

### 3.4 Structure validity

The sample adequacy and data suitability for factor analysis were supported by KMO = 0.868 and Bartlett’s test of sphericity *χ*^2^ = 2080.882 (*p* < 0.001). An initial EFA using PAF with the Kaiser criterion (eigenvalues > 1) yielded a five-factor solution, accounting for 52.23% of the total variance, with factor intercorrelations ranging from −0.022 to 0.574 (Supplementary Table S2). However, factor 5 comprised only two items (items 6 and 7), and item 9 failed to meet the threshold (pattern coefficients < 0.30). Given that the conventional scree plot indicated a substantial inflection after the second factor (Supplementary Figure S1), a fixed two-factor solution was subsequently evaluated, explaining 39.68% of the total variance, with a strong factor correlation of 0.547. However, this solution exhibited poor item retention, with six items (items 2, 4, 5, 6, 10, and 13) demonstrating primary pattern coefficients below 0.30 (Supplementary Table S3). To empirically establish the optimal number of factors, a parallel analysis was performed prior to extraction, which strongly supported a four-factor solution (Figure 1). This solution was also consistent with the original WEPS theoretical four-dimensional structure. Consequently, a PAF with a fixed four-factor extraction and Promax oblique rotation was conducted (Table 2). The four-factor solution accounted for 49.01% of the total variance, with factor intercorrelations ranging from 0.155 to 0.547, supporting the appropriateness of the oblique rotation. All 20 items had primary pattern coefficients ≥ 0.30 in the pattern matrix. Overall, the four-factor solution was retained due to its support from parallel analysis, an interpretable factor structure, and conceptual alignment with the theoretical framework. Specifically, factor 1, labeled “Intention”, included items 1, 8, 16, 17, 18, 19, and 20 and reflected students’ intention to pursue work in geriatric care; factor 2, labeled “Perceived Behavioral Control”, comprised items 9, 11, 12, 13, 14, and 15 and captured students’ perceived competence, skills, and educational guidance relevant to engaging in geriatric care. Factor 3, labeled “Attitude”, comprised items 2, 3, 4, 5, and 10, which reflected students’ positive or negative affective evaluations of working with older adults. Finally, factor 4, labeled “Subjective Norm”, consisted of items 6 and 7 and reflected students’ perceived social expectations and normative influences regarding working with older adults.

**Figure 1:**
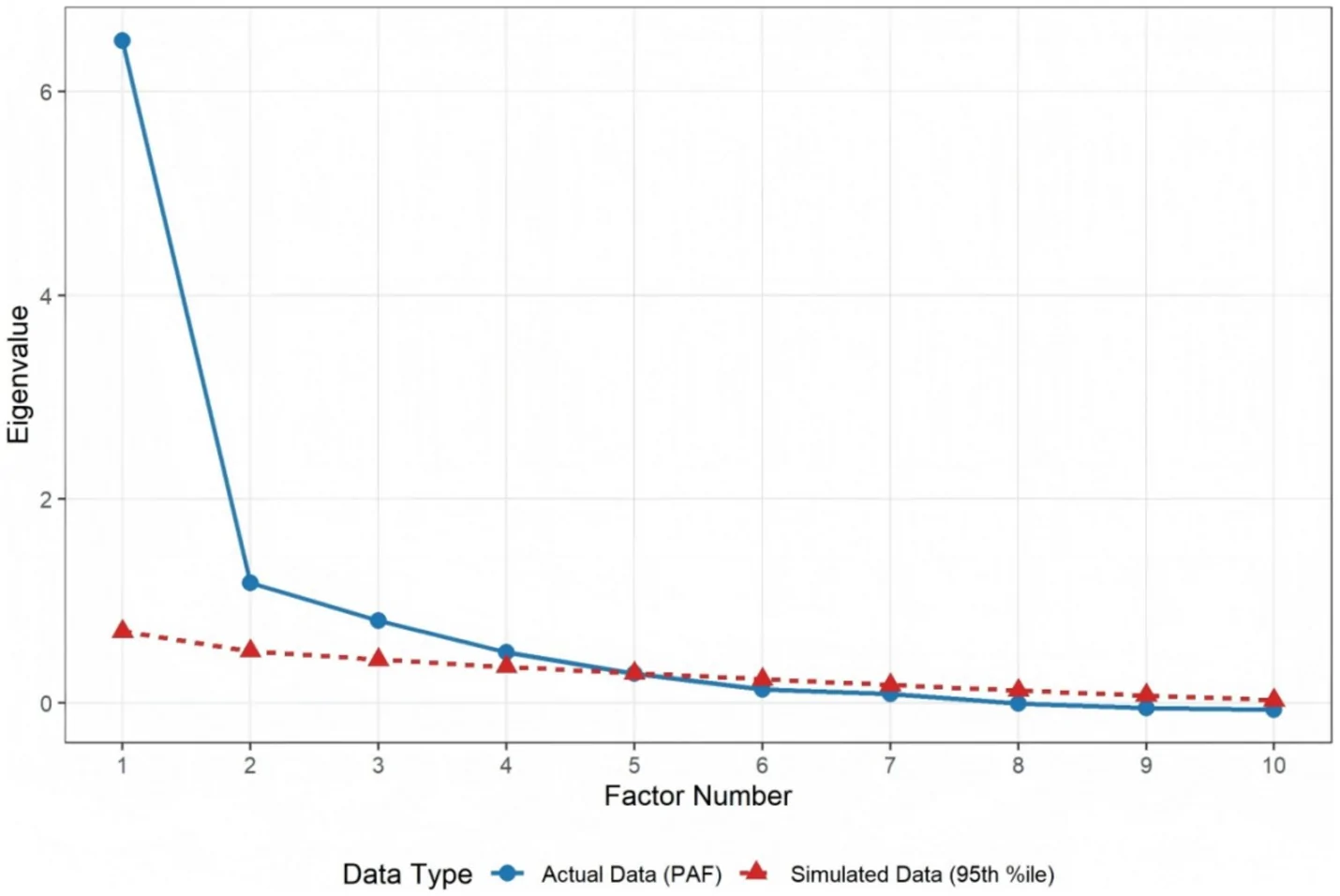
Scree plot of the parallel analysis (*N* = 207).

### 3.5 Convergent validity

Pearson’s correlation analyses showed that the WEPS-D total score showed a lower correlation with the KAOP total score (*r* = 0.385, *p* < 0.01), which did not meet the prespecified criterion (*r* ≥ 0.40). However, factor 3 (“Attitude”) demonstrated a moderate correlation with the KAOP (*r* = 0.515, *p* < 0.01), providing evidence of convergent validity at the factor level. Regarding AS, the strongest correlation with the WEPS-D total score was observed for “Appearance” (*r* = 0.344, *p* < 0.01). For FS, correlations with the WEPS-D total score were generally weak across the different domains, suggesting a more limited relationship between future self-related expectations and willingness to work with older adults (Table 3).

**Table 3:** Correlations between the WEPS-D and other variables.

Scale		WEPS (20 items)	Factor 1 (7 items)	Factor 2 (6 items)	Factor 3 (5 items)	Factor 4 (2 items)
Mean (SD)		87.65 (15.04)	26.10 (8.86)	27.76 (4.76)	25.15 (3.25)	8.65 (2.22)
KAOP		0.385^**^	0.332^**^	0.219^**^	0.515^**^	0.055
AS	Family	0.108	0.109	0.080	0.052	0.051
	Friendships	0.201^**^	0.170	0.174^*^	0.114	0.142^*^
	Leisure	0.088	0.091	0.094	0.142^*^	−0.178^*^
	Personality	0.060	0.023	0.111	0.099	−0.070
	Finances	0.068	−0.070	0.075	0.069	−0.077
	Work	0.194^**^	0.188^**^	0.085	0.207	0.078
	Appearance	0.344^**^	0.346^**^	0.175^*^	0.295^**^	0.144^*^
	Fitness	0.123	0.088	0.163^*^	0.151^*^	−0.090
	Health	0.174	0.235^**^	0.104	0.052	−0.056
FS	Family	0.105	0.032	0.204^**^	0.087	0.020
	Friendships	0.020	−0.029	0.110	0.022	−0.016
	Leisure	0.000	−0.084	0.122	0.052	−0.003
	Personality	0.053	−0.035	0.138^*^	0.125	0.021
	Finances	0.036	−0.023	0.123	0.069	−0.026
	Work	0.116	0.075	0.166	0.071	0.027
	Appearance	0.218^**^	0.162	0.227^**^	0.143	0.133
	Fitness	0.116	0.050	0.162^*^	0.114	0.069
	Health	0.125	0.070	0.200	0.092	0.007

*p < 0.05; **p < 0.01, AS: Age Stereotypes, FS: Future Self-views, KAOP: Kogan Attitudes toward Older People Scale, WEPS: Willingness to Work with Elderly People Scale.

### 3.6 Test–retest reliability

A total of 52 nursing students completed the retest assessment, representing 25.1% of the initial sample (*n* = 207). The mean baseline WEPS-D score was 83.88 (SD = 12.35) among retest completers and 88.91 (SD = 15.68) among non-completers. The difference between the two groups was statistically significant (*t* = −2.102, *p* = 0.037). The ICC for the WEPS-D was 0.882 (95% CI: 0.803 to 0.930), indicating that the reliability of the scale was high.

### 3.7 Internal consistency

The internal consistency of the WEPS-D was good (Cronbach’s α = 0.894, McDonald’s ω = 0.902) (Table 4). For the sub-factors, Cronbach’s α ranged from 0.580 to 0.919, while McDonald’s ω ranged from 0.653 to 0.931. McDonald’s ω was not applicable to factor 4 because model identification requires a minimum of three items per factor. However, factor 4 showed a Spearman–Brown coefficient of 0.586.

**Table 4:** Internal consistency of the WEPS-D and its sub-factors.

Internal consistency	WEPS (20 items)	Factor 1 (7 items)	Factor 2 (6 items)	Factor 3 (5 items)	Factor 4 (2 items)
Cronbach’s α	0.894	0.919	0.728	0.643	0.580
McDonald’s ω	0.902	0.931	0.722	0.653	NA
Spearman-Brown	NA	NA	NA	NA	0.586

NA: not applicable.

### 3.8 Floor and ceiling effects

The proportions of participants achieving the lowest and highest possible scores were 1 and 0.5%, respectively.

### 3.9 Correlation analyses

A univariate analysis was conducted for the following variables: gender, age, country, education, marital status, presence of older adults living in the neighborhood, having grandparents, previous experience working with older adults, previous visits to nursing homes or geriatric clinics, attitude toward aging, and subjective age perception. The results showed that nursing students who were mature students, completed vocational training, and held a positive attitude toward aging had significantly higher WEPS-D scores. For neighborhood composition, the results showed a significant overall difference in WEPS-D scores among students who reported no, only a few, or several older adults living in their neighborhood (*F* = 3.890, *p* = 0.022). However, none of the Bonferroni-adjusted pairwise comparisons reached statistical significance (Table 1).

Age, education, neighborhood, and attitude were subsequently entered into a multiple linear regression model (Table 5). The regression model explained 20.6% of the variance in the WEPS-D score (*R*^2^ = 0.206), with an adjusted R^2^ of 0.182. No serious multicollinearity was detected among the independent variables (tolerance = 0.789–0.959, VIF = 1.043–1.267). In the regression model, age and neighborhood were not independently associated with WEPS-D scores. However, education (*B* = −6.829, *β* = −0.221, 95% CI: −10.857 to −2.801, *p* < 0.001) and attitude toward aging (Negative vs. Positive: *B* = −13.732, *β* = −0.264, 95% CI: −20.698 to −6.766, *p* < 0.001; Neutral vs. Positive: *B* = −9.230, *β* = −0.307, 95% CI: −13.437 to −5.024, *p* < 0.001) were significantly associated with WEPS-D scores. Specifically, students with higher education had lower WEPS-D scores than those with vocational training. In contrast, students with negative or neutral attitudes toward aging had lower WEPS-D scores than those with positive attitudes, indicating a lower willingness to work with older adults.

**Table 5:** Multiple linear regression model.

Predictor	B	SE	*β*	95% CI		*t*	*p*	VIF
				Lower	Upper			
Age	0.205	0.104	0.135	−0.001	0.410	1.960	0.051	1.198
Education	−6.829	2.043	−0.221	−10.857	−2.801	−3.343	**<0.001**	1.101
Neighborhood (no vs. yes, several)	−5.915	4.306	−0.088	−14.406	2.576	−1.374	0.171	1.043
Neighborhood (yes, only a few vs. Yes, several)	−2.096	2.204	−0.062	−6.441	2.250	−0.951	0.343	1.071
Attitude (negative vs. positive)	−13.732	3.533	−0.264	−20.698	−6.766	−3.887	**<0.001**	1.163
Attitude (neutral vs. positive)	−9.230	2.133	−0.307	−13.437	−5.024	−4.327	**<0.001**	1.267

Age was entered as a continuous variable. Education was dummy-coded as vocational training = 0 and higher education = 1, with vocational training as the reference category. Neighborhood experience was represented by two dummy variables: Neighborhood (No vs. Yes, several), coded 1 = No and 0 = Yes, several; and Neighborhood (Yes, only a few vs. Yes, several), coded 1 = Yes, only a few and 0 = Yes, several. Attitude toward aging was represented by two dummy variables: Attitude (Negative vs. Positive), coded 1 = Negative and 0 = Positive; and Attitude (Neutral vs. Positive), coded 1 = Neutral and 0 = Positive. “Yes, several” was the reference category for neighborhood experience, and “Positive” was the reference category for attitude toward aging. Bold values indicate statistically significant results (*p* < 0.05).

Descriptive analyses of the multiple-choice items indicated that higher WEPS-D scores were observed among nursing students whose closest grandparents lived in the same household, who saw their grandparents daily, who considered a person old from the age of 90 years, who could imagine working in a nursing home, geriatric care, or geriatric medicine in the future, who most preferred working with older adults, who identified abuse and discrimination as a major concern regarding aging, and who reported that their grandmother had the greatest influence on their view of old age (Table 1).

## 4 Discussion

This study aimed to culturally adapt and evaluate the psychometric properties of the WEPS-D and to explore factors associated with nursing students’ willingness to work with older adults. The findings demonstrated that the WEPS-D is a reliable and valid instrument for use among German-speaking nursing students. In addition, vocational training and positive attitudes toward aging were independently associated with greater willingness to work with older adults. These findings are particularly relevant in the context of increasing workforce shortages in geriatric care and highlight the potential role of nursing education in fostering future interest in geriatric nursing careers.

The psychometric evaluation supported a four-factor structure consisting of “Intention”, “Perceived Behavioral Control”, “Attitude”, and “Subjective Norm”, which was consistent with the theoretical structure of the original WEPS and the Turkish version (Ajzen, 1991; Akpınar Söylemez et al., 2022; Momtaz et al., 2018). However, although the four-factor structure was retained, four items (items 1, 8, 9, and 10) were assigned to different factors in the German-speaking sample than in the original WEPS. This finding suggests that the overall theoretical framework of willingness to work with older people was relatively stable across populations, while the interpretation of individual items may vary according to cultural and educational contexts. Compared with the original WEPS, factor 1 additionally included items 1 and 8, which were originally classified under “Attitude” and “Subjective Norm”, respectively. Item 1 (“Working with the elderly is gratifying”) theoretically reflects an affective evaluation of working with older people. In the German-speaking nursing student sample, this kind of gratification may also be more closely linked to an individual’s positive tendency to engage in geriatric care in the future, which may explain why it loaded on the “Intention” factor. Item 8 (“There is enough encouragement for working with the elderly”) may have been understood by German-speaking nursing students as reflecting sufficient encouragement to pursue geriatric care, rather than the expectation of others that they should engage in such work. In this context, encouragement may be perceived as promoting students’ own willingness to pursue geriatric care, which may explain why the item loaded on the “intention” factor rather than the “Subjective Norm” factor. Factor 2 additionally included item 9 (“My professors advise me to consider a career in elderly care”), which was originally classified under “Subjective Norm” because it explicitly refers to advice from professors. In the German-speaking educational context, students may interpret such advice not simply as social pressure or expectations but as guidance that helps them evaluate their preparedness and feasibility of entering geriatric care. This interpretation is consistent with the broader meaning of perceived behavioral control. Factor 3 also included item 10 (“Working with elderly people is socially valuable”), which was originally classified under “Subjective Norm” but loaded on “Attitude” in the present study. This reassignment is conceptually plausible because the statement evaluates the desirability of working with older adults, rather than explicitly assessing perceived expectations or approval from significant others. In the German-speaking nursing student sample, students may have interpreted the social value of geriatric work primarily as a personal evaluation, which is more consistent with the evaluative nature of the “Attitude” factor. Factor 4 retained items 6 and 7, consistent with their classification in the original WEPS, suggesting that the normative component of the WEPS was recognizable in the German-speaking sample. However, factor 4 showed relatively low internal consistency (Cronbach’s α = 0.580; Spearman–Brown coefficient = 0.586), which was similar to that reported for the corresponding dimension in the original WEPS (Momtaz et al., 2018). In the present study, the two-item factor provides less information about the breadth of the latent construct and may yield a less stable reliability estimate than factors represented by a larger number of items (Eisinga et al., 2013). Therefore, the “Subjective Norm” dimension should be interpreted cautiously.

The WEPS-D showed a positive association with attitudes toward older adults measured by the KAOP, but the correlation did not meet the prespecified criterion for convergent validity. The lower magnitude of this correlation is theoretically meaningful because willingness to work with older adults represents a construct that is related to, but distinct from, general attitudes toward aging (Zhang and Zhou, 2020). According to the theory of planned behavior (Ajzen, 1991), attitudes are important antecedents of behavioral intentions but do not fully overlap with them. Therefore, the distinction in our study further supports the conceptual differentiation between attitudinal evaluations and behavioral readiness, indicating that the WEPS-D captures a more action-oriented construct. Notably, factor 3 of the WEPS-D showed a correlation with the KAOP that exceeded the prespecified threshold of 0.40 (*r* = 0.515, *p* < 0.01). One possible explanation is that several items within this factor (e.g., items 2, 3, 4, 5, and 10) reflect value-based evaluations of working with older adults. These items are more directly aligned with value-based attitudes, which may increase conceptual overlap with the KAOP. Regarding AS, correlations were generally small to moderate, with “Appearance” showing the strongest association. Physical appearance constitutes one of the most salient and immediately observable characteristics of older adults, as perceptions of age are often formed on the basis of visible characteristics such as wrinkles, gray hair, and facial morphology (Richeson and Shelton, 2006). This may explain why the “Appearance” domain (“Older adults have an unattractive/attractive appearance”) showed a relatively stronger association with the WEPS-D compared to other, less directly observable stereotype domains, such as “Finances” or “Family” roles. Positive evaluations of appearance-related characteristics may contribute to broader positive perceptions of older adults, which in turn may be reflected in greater willingness to work with this population. In addition, FS showed consistently weak correlations with the WEPS-D, suggesting that self-related aging expectations represent a distal cognitive construct with limited conceptual proximity to occupational willingness. According to the concept of possible selves (Markus and Nurius, 1986), future self-concepts are internal cognitive representations of possible future identities that primarily function as motivational and self-regulatory guides, rather than being directly translated into specific behavioral intentions in professional contexts.

This study examined factors related to nursing students’ willingness to work with older adults. Greater willingness to work with older adults was independently associated with vocational training and positive attitudes toward aging. Specifically, students with higher education had significantly lower WEPS-D scores than those with vocational training, indicating that students with vocational training reported greater willingness to work with older adults. This finding may reflect the importance of early practical exposure and experiential learning. It may be explained by the structure of vocational education and training systems in German-speaking countries. According to the Organisation for Economic Co-operation and Development framework (Organisation for Economic Co-operation and Development (OECD), 2025), vocational education and training were primarily delivered through a dual-system model that integrates school-based theoretical instruction with structured, company-based training. This combination may facilitate learning both in educational institutions and in real workplace environments, particularly in clinically relevant settings such as hospitals, long-term care facilities, and community care services. The development of practical competencies and professional identity through workplace learning may foster a stronger sense of preparedness and commitment to caring roles (Klotz et al., 2014), which may be associated with a greater willingness to work with older adults. Educational experiences that allow students to interact meaningfully with older adults may help reduce stereotypes, increase confidence, and strengthen interest in geriatric careers. In the present study, students with negative or neutral attitudes toward aging had significantly lower WEPS-D scores than those with positive attitudes, supporting an association between positive attitudes toward aging and greater willingness to work with older adults. This finding was consistent with previous studies conducted in Israel, Vietnam, China, and Türkiye (Birimoglu Okuyan et al., 2020; Liu et al., 2026; Thuong et al., 2025; Vitman-Schorr and Rozani, 2025). It is also supported by the theory of planned behavior, which proposes that attitudes toward a behavior are among the most important determinants of behavioral intentions (Ajzen, 1991). Importantly, attitudes are modifiable through education. Previous studies have demonstrated that intergenerational learning experiences, contact-based interventions, and gerontological education can improve students’ perceptions of aging (Hand et al., 2025). From an educational perspective, the findings suggest that willingness to work with older adults should not be regarded as a fixed personal characteristic. Instead, willingness appears to be influenced by educational experiences and attitudes toward aging. Nursing programs may therefore play an important role in strengthening students’ interest in geriatric careers through targeted educational strategies, clinical placements, and intergenerational learning opportunities.

For the multiple linear regression model, the small difference between R^2^ and adjusted *R*^2^ indicates that the model was not substantially overfitted. The low VIF values confirmed the absence of multicollinearity, supporting the robustness of the regression estimates (Hair et al., 2019). However, the model explained a modest proportion of variance in WEPS (*R*^2^ = 0.206; adjusted *R*^2^ = 0.182). This suggests that willingness to work with older adults is likely influenced by a broader range of factors not included in the present model (Hair et al., 2010). Several variables in the present study were collected using multiple-response items and could not be included in the regression analysis, which may be the reason for reducing the overall explanatory power of the model.

Beyond the regression findings, descriptive analyses indicated that students who reported living with grandparents or having frequent contact with older family members tended to report greater willingness. As these variables were not included in the multivariable model, this finding should be interpreted as an exploratory observation rather than as an independently established group difference. Nevertheless, the finding is consistent with previous studies suggesting the importance of intergenerational relationships in shaping willingness to engage in geriatric care (Thuong et al., 2025; Tunç et al., 2026). This finding may also be interpreted in light of the social projection process (Clement and Krueger, 2002), whereby individuals may draw on their own experiences to form generalized perceptions of others. In this context, frequent or close intergenerational contact may provide students with more positive and familiar experiences of older adults (Vitman-Schorr and Rozani, 2025), which may in turn shape their perceptions of aging and responses toward older people. Such experiences may contribute to greater willingness to consider working with older adults. This finding highlights the potential value of meaningful intergenerational contact in encouraging future nurses to consider careers involving older adults.

Taken together, the present findings suggest that willingness to work with older adults should not be viewed solely as an individual preference. Rather, willingness appears to be shaped by educational experiences, attitudes toward aging, and opportunities for meaningful contact with older adults. This perspective is particularly important given the growing demand for qualified professionals in geriatric care. Nursing schools may therefore benefit from integrating more gerontological content, expanding clinical placements in elderly care settings, and implementing educational strategies specifically designed to increase students’ interest in working with older adults.

### 4.1 Limitations

The present study also has several limitations. First, due to the sample size, confirmatory factor analysis (CFA) was not conducted to further validate the four-factor structure of the WEPS-D. Future studies should recruit larger samples of German-speaking nursing students to enable robust CFA and further examine the factorial validity of the scale. Second, the majority of participants were female nursing students, which likely reflects the gender distribution within nursing education. This gender imbalance may limit the generalizability of the findings. In addition, convenience sampling and voluntary participation may have introduced potential self-selection bias. This was also evident in the test–retest analysis, as baseline WEPS-D scores differed significantly between retest completers and non-completers, suggesting that the retest subsample may not have been fully representative of the initial sample. In addition, as 98.6% of participants reported previous work experience with older adults, students with prior exposure to older adults may have been overrepresented, which may further limit the representativeness of the findings. Third, because the total number of institutions approached and the number of students who received the survey invitation were not systematically recorded, a response rate could not be calculated, and the extent of potential non-response bias could not be assessed. Fourth, given the cross-sectional nature of the study, causal relationships between the variables cannot be inferred. Although we identified differences across sociodemographic characteristics, longitudinal studies are needed to better understand how willingness to work with older adults develops and changes over time. Fifth, the four-factor solution explained 49.01% of the total variance, which was relatively modest. Nevertheless, the four-factor solution was supported by parallel analysis and was consistent with the theoretical four-dimensional structure of the original WEPS, providing support for retaining this solution despite the relatively modest explained variance. Sixth, no adjustment for multiple testing was conducted in the correlation analysis (Table 3). Therefore, the possibility of an inflated Type I error rate cannot be excluded (Albers, 2019). The correlations reported should be interpreted as exploratory findings and should be confirmed in future studies using appropriate adjustments for multiple comparisons. Finally, this study primarily employed a quantitative design to explore factors associated with willingness to work with older adults. However, willingness is likely shaped by multiple and interacting influences that may not be fully captured by the present model. Future research would benefit from a qualitative study, including interviews with students from different educational levels and training backgrounds, to gain deeper and more comprehensive insight into the factors driving willingness to work with older adults.

### 4.2 Relevance for research practice

The WEPS-D may serve as a useful tool for identifying nursing students’ preferences and potential career orientations related to working with older adults. By distinguishing levels of willingness across different subgroups, the scale can help researchers and educators better understand which students are more or less inclined to pursue geriatric-related career paths. This may contribute to early identification of workforce tendencies. In addition, the identified associations between WEPS-D scores and sociodemographic characteristics highlight the importance of targeted educational strategies. Nursing schools and educators may use these findings to design tailored interventions aimed at enhancing students’ willingness to work with older adults and ultimately support evidence-based planning for geriatric care workforce development.

## 5 Conclusion

The WEPS-D consists of 20 items across four dimensions: “Intention”, “Perceived Behavioral Control”, “Attitude”, and “Subjective Norm”. The scale demonstrated generally acceptable psychometric properties among German-speaking nursing students, including a clear four-factor structure, strong content validity, and excellent test–retest reliability. Convergent validity was supported for factor 3, whereas the correlation between the WEPS-D and the KAOP did not meet the prespecified criterion. Internal consistency was good at the total-scale level but lower for factors 3 and 4. Beyond its psychometric properties, the WEPS-D may support nursing educators in identifying students less inclined to consider geriatric careers and in developing targeted educational strategies to strengthen the future geriatric nursing workforce. Furthermore, higher education was associated with lower willingness to work with older adults than vocational training, while negative and neutral attitudes toward aging were associated with lower willingness compared with positive attitudes. The WEPS-D may therefore provide a valuable resource for monitoring nursing students’ willingness to work with older adults and informing educational initiatives aimed at strengthening the future geriatric workforce.

## Data Availability

The datasets presented in this study can be found in online repositories. The names of the repository/repositories and accession number(s) can be found at: open Science Framework (https://osf.io/m5f7b/overview).

## Footnotes

1
https://osf.io/m5f7b/overview

2
https://www.soscisurvey.de/de/index
