# Supplementary material for: Identifying future geriatric nurses: psychometric evaluation of the German Willingness to Work with Elderly People Scale among nursing students
Source: Front Psychol. 2026 Oct 15;17:1933160. doi: 10.3389/fpsyg.2026.1933160 (PMC13716665; doi:10.3389/fpsyg.2026.1933160)
Supplement: Supplementary file 3 [file Table_2.DOCX]

**Supplementary Table S2: Initial five-factor solution based on the Kaiser criterion**

| **Items** | | **Factor 1** | **Factor 2** | **Factor 3** | **Factor 4** | **Factor 5** |
| --- | --- | --- | --- | --- | --- | --- |
| 1 | Working with the elderly is gratifying | 0.489 |  |  |  |  |
| 2 | Care of elderly patients is the waste financial resources^#^ |  |  | 0.579 |  |  |
| 3 | Working with aged patients is disappointing^#^ |  |  | 0.455 |  |  |
| 4 | Working with older adults is a dead-end job^#^ |  |  | 0.521 |  |  |
| 5 | Working with the elderly is a highly rewarding experience |  |  |  | 0.388 |  |
| 6 | Caring for the elderly is a human duty |  |  |  |  | 0.541 |
| 7 | My culture encourages me to work with the elderly |  |  |  |  | 0.752 |
| 8 | There is enough encouragement for working with the elderly | 0.563 |  |  |  |  |
| 9 | My professors advise me to consider aged care career |  |  |  |  |  |
| 10 | Working with elderly people is socially valuable |  |  |  | 0.431 |  |
| 11 | I achieve competencies for the care of older patients |  |  |  | 0.658 |  |
| 12 | I have professional competencies in aged care |  | 0.791 |  |  |  |
| 13 | In the curriculum there is enough aged care training |  |  |  | 0.316 |  |
| 14 | I have the skills to work with the elderly |  | 0.536 |  |  |  |
| 15 | I have the necessary capabilities to provide end-of-life care |  | 0.744 |  |  |  |
| 16 | One of my career priorities upon graduation is aged care | 0.795 |  |  |  |  |
| 17 | I will never consider an aged care job^#^ | 0.593 |  |  |  |  |
| 18 | After graduation, I would like to work in community aged care | 0.947 |  |  |  |  |
| 19 | Upon graduation, I would prefer to work with the elderly | 1.017 |  |  |  |  |
| 20 | After graduation, I definitely choose working with older people | 0.962 |  |  |  |  |

Note: Blank cells indicate factor loadings < 0.30 in the pattern matrix.; ^#^Items 2, 3, 4, and 17 were reverse-scored before calculating item-total correlations and factor loadings.
