# Supplementary material for: Identifying future geriatric nurses: psychometric evaluation of the German Willingness to Work with Elderly People Scale among nursing students
Source: Front Psychol. 2026 Oct 15;17:1933160. doi: 10.3389/fpsyg.2026.1933160 (PMC13716665; doi:10.3389/fpsyg.2026.1933160)
Supplement: Supplementary file 4 [file Table_3.DOCX]

**Supplementary Table S3: Fixed two-factor solution based on the scree plot**

| **Items** | | **Factor 1** | **Factor 2** |
| --- | --- | --- | --- |
| 1 | Working with the elderly is gratifying | 0.515 | 0.320 |
| 2 | Care of elderly patients is the waste financial resources^#^ |  |  |
| 3 | Working with aged patients is disappointing^#^ | 0.425 |  |
| 4 | Working with older adults is a dead-end job^#^ |  |  |
| 5 | Working with the elderly is a highly rewarding experience |  |  |
| 6 | Caring for the elderly is a human duty |  |  |
| 7 | My culture encourages me to work with the elderly | 0.503 |  |
| 8 | There is enough encouragement for working with the elderly | 0.669 |  |
| 9 | My professors advise me to consider aged care career | 0.368 |  |
| 10 | Working with elderly people is socially valuable |  |  |
| 11 | I achieve competencies for the care of older patients |  | 0.429 |
| 12 | I have professional competencies in aged care |  | 0.804 |
| 13 | In the curriculum there is enough aged care training |  |  |
| 14 | I have the skills to work with the elderly |  | 0.735 |
| 15 | I have the necessary capabilities to provide end-of-life care |  | 0.641 |
| 16 | One of my career priorities upon graduation is aged care | 0.773 |  |
| 17 | I will never consider an aged care job^#^ | 0.492 |  |
| 18 | After graduation, I would like to work in community aged care | 0.995 |  |
| 19 | Upon graduation, I would prefer to work with the elderly | 0.941 |  |
| 20 | After graduation, I definitely choose working with older people | 0.929 |  |

Note: Blank cells indicate factor loadings < 0.30 in the pattern matrix.; ^#^Items 2, 3, 4, and 17 were reverse-scored before calculating item-total correlations and factor loadings.
