# Supplementary material for: Identifying future geriatric nurses: psychometric evaluation of the German Willingness to Work with Elderly People Scale among nursing students
Source: Front Psychol. 2026 Oct 15;17:1933160. doi: 10.3389/fpsyg.2026.1933160 (PMC13716665; doi:10.3389/fpsyg.2026.1933160)
Supplement: Supplementary file 5 [file Supplementary_file_1.DOCX]

**Supplementary Figure S1: Conventional scree plot**
